# An End-to-End Automated License Plate Recognition System Using YOLO Based Vehicle and License Plate Detection with Vehicle Classification

**DOI:** 10.3390/s22239477

**Published:** 2022-12-04

**Authors:** Reda Al-batat, Anastassia Angelopoulou, Smera Premkumar, Jude Hemanth, Epameinondas Kapetanios

**Affiliations:** 1 School of Computer Science and Engineering, University of Westminster, London W1W 6UW, UK; 2 Karunya Institute of Technology and Sciences, Karunya University, Coimbatore 641114, India; 3 School of Physics, Engineering and Computer Science, University of Hertfordshire, Hatfield AL10 9EU, UK

**Keywords:** automatic license plate recognition, convolutional neural networks, YOLO

## Abstract

An accurate and robust Automatic License Plate Recognition (ALPR) method proves surprising versatility in an Intelligent Transportation and Surveillance (ITS) system. However, most of the existing approaches often use prior knowledge or fixed pre-and-post processing rules and are thus limited by poor generalization in complex real-life conditions. In this paper, we leverage a YOLO-based end-to-end generic ALPR pipeline for vehicle detection (VD), license plate (LP) detection and recognition without exploiting prior knowledge or additional steps in inference. We assess the whole ALPR pipeline, starting from vehicle detection to the LP recognition stage, including a vehicle classifier for emergency vehicles and heavy trucks. We used YOLO v2 in the initial stage of the pipeline and remaining stages are based on the state-of-the-art YOLO v4 detector with various data augmentation and generation techniques to obtain LP recognition accuracy on par with current proposed methods. To evaluate our approach, we used five public datasets from different regions, and we achieved an average recognition accuracy of 90.3% while maintaining an acceptable frames per second (FPS) on a low-end GPU.

## 1. Introduction

The volume of motor traffic is increasing day by day on the roadways, and it is essential to improve the traffic management system by ensuring road safety, traffic efficiency and mobility in a reliable way. An ALPR system is a fully automated, high- speed camera-based system that tracks, records, and reports vehicle license plates. Advancements in ALPR technology and the wide adoption of deep learning networks can improve the existing systems and also maximize the operational efficiency of ITS systems. It is also used in the commercial industry for parking management [1], automated toll collection [2], security, and surveillance [3,4].

A state-of-the-art ALPR system consists of three main stages: vehicle detection (VD), license plate detection (LPD), and license plate recognition (LPR). Additionally, a classification process is also performed to identify the type of vehicle which can be easily expanded on for the vehicle make, model, year and more to make it more useful to the overall system. It is critical to identify an emergency vehicle, such as an ambulance or fire services, and allow them to pass without issuing traffic tickets or fines. The whole process starts with the image data source, for example, a CCTV camera overlooking a motorway. The images obtained from the camera first pass through the VD stage, where the vehicle patches are obtained. Then, each vehicle patch goes through the LPD stage to get the LP patch, followed by the LPR stage to detect all characters and recognize the LP text to identify the vehicle.

It is very important to consider all three stages because each one affects the performance of the next. For example, starting off with the vehicle patches gives you a great advantage, as you can assume 100% accuracy for the vehicle detection stage. This also applies to the LP detection stage: starting with the LP patch allows you to assume that you have 100% detection accuracy on the two previous stages, which eliminates a considerable amount of variability that would otherwise be present in your practice and your final result will not reflect it. This “skipping” of stages will not be reflected in the recognition accuracy, hence why it is critical to include all stages of the pipeline in an ALPR system.

The majority of previous works on the ALPR pipeline use a pre-defined rules and/or post processing steps. Each country may have different LP layouts and, thus, the positions of letters and digits on the LP changes. For example, vehicles entering from one country into another may have a different LP layout, and some may have a personalized LP. Such scenarios have a completely different LP layout from what is normally used in the reference country. So, any pre-defined rules that may have been setup to increase the recognition rate will most likely fail in those cases. Furthermore, if this is a known issue, it can be exploited and cause further problems, so using any specific prior knowledge is not the way forward.

In this paper, we propose a fully automated ALPR pipeline that does not use any pre-defined rules, uses a wide range of datasets that have different character sequences and conditions, and increases the datasets by more than three times by using various data augmentation and data generation techniques coupled with the You Only Look Once (YOLO) detector at each stage. There are three main stages that comprise the whole ALPR system. The first stage is the vehicle detection stage, where all the vehicles in the image are detected. Following the vehicle detection stage, each vehicle patch is cropped and fed into an LP detector, which detects the LP of the vehicle. Since each vehicle can only have one LP, the detection with the highest confidence is chosen if multiple detections occur. Additionally, for each vehicle patch after the vehicle detection stage, each vehicle patch goes through a ResNet50 classifier [5] to classify the vehicles into three classes: trucks, emergency vehicles, and others. The final stage is the LP character detection stage, where each character of the LP is detected and assembled to form the full LP text. The major contributions of this paper are as follows:

- A streamlined, generalizable ALPR pipeline
- A fully automated ALPR system that does not require any pre-defined rules or post-processing steps.
- A customized data augmentation technique and data generation to synthesize new license plates to increase data
- An elementary vehicle classifier that can be expanded on
- A methodological analysis of the proposed method with preceding works in literature.
- In addition, we have evaluated our ALPR system with five datasets from five different regions of the world, so we can show the generalizability of the proposed work and also in real-world applications such as different lighting conditions, backgrounds, and orientations.

The paper is structured as follows: Section 2 gives an overview of the related works. Section 3 outlines the methodological approach, and Section 4 explains the experimental design and results. Finally, we conclude and confer a summary of the key contributions and results of this paper.

## 2. Related Work

This section explores some relevant works on ALPR system and its challenges in methodology. An ALPR workflow includes mainly three stages: vehicle detection, license plate detection, and license plate recognition. 

Previous works have validated their results by considering one or two stages of the ALPR system. Examples of this are [6,7] where the first two stages, the VD and the LPD stages, were skipped. In particular, one of the studies [7] only considered the LPD stage and focused on obtaining the angle of the LP bounding box (BB). Since it achieved great detection results, it simplified the problem by forcing their ALPR system to output only one BB per image (only one vehicle for every image), which is not practical, especially for a general ALPR system. Similarly, the VD stage was not considered in some studies [8,9] but the LPD and LPR stages were performed. This might be just because of what was needed for their specific application; however, the full pipeline is needed for a complete automated ALPR system. Likewise, Refs. [9,10] consider only one LP per image, thus one vehicle per frame; this is not practical in real-time scenarios. As multiple vehicle images are bound in a single frame, it may have a chance to increase the processing time. However, this may be useful for parking spaces, where the conditions are very fixed and only one vehicle is present at the entrance gate, but it is not suitable for a real-world general framework on roadways. Even though the processing of these three stages is not convenient, the final accuracy depends on the relative contribution of all three stages. To obtain a completely automated system, we should have to process the whole three stages.

Some relevant works in literature consider the whole pipeline, such as [11,12] and achieve great results. However, Ref. [11] only considers one dataset with Brazilian LPs, where the images were only frontal views of the vehicles. In the literature, most works authenticate their results using no more than three datasets, which biases the generalizability of the method [8,9]. Subsequently, Ref. [12] achieved very good results on previous methods and compared their method on eight public datasets. Despite the fact that their results were significant, they relied on exploiting specific country layouts, and were limited to particular set of rules based on a country associated with that LP layout. For example, it works only if the first two characters of the LP are characters. If a LP is detected with the digit “1”, the system would consider it a character “I” instead of a digit, and it may have a huge impact on the final result. Thus, the generalizability of this method is poor and limited on roadways with a distinct LP layout. In our work, we make use of different country layouts, and our findings are promising for a universally pertinent ALPR system.

Having an ALPR system that performs in real-time is very important. This is because if a vehicle is travelling at, for example, 60 miles per hour and you have a low FPS, the vehicle might only be in the frame once or twice, and depending on the camera location and how many vehicles are in the frame and their speeds, vehicles might be completely missed. Thus, having an ALPR system that performs at a relatively high FPS is important not only to ensure all vehicles are detected but also to allow the system more attempts to detect and recognize the LP as the vehicle moves across the frame, where each position will present different lighting conditions, camera angles, backgrounds, etc.

Examples of where the FPS was too low to use in practical settings are [8,10]. Despite Ref. [10] using a dedicated GPU (GT-740M), it performed very slowly at 230 ms (4 FPS) to only detect LPs, which is way too slow for real-world applications with high-speed moving vehicles. A previous study [8] achieved relatively good results, but on a very high-end GPU (NVIDIA Tesla K40c), they had multiple steps, using high-demanding methods such as sliding windows, causing their system to operate at two seconds per frame, which is not practical.

There are some works that achieve great FPS, such as [7,9,11,12], which achieve 76 FPS using a high-end GPU, but the recognition accuracy is very poor on the SSIG- SegPlate dataset, at 63%. Ref. [7] achieved a very good ∼5 ms per frame on a relatively inexpensive GPU (FTX980) for the LPD stage, which is the only stage they considered and is not really comparable to the overall FPS of the above methods. One study [9] also used a relatively inexpensive GPU (NVIDIA 1080 Ti) and was able to process images in 0.0443 s for both the LPD and LPR stages, but they only considered one LP per image. Another study [12] achieved 73 FPS with a high-end GPU (NVIDIA Titan XP) when one vehicle is present in the image, but when five vehicles are present, the FPS drops to 29 FPS, which is still good. This is because they are using the YOLOv2 detector, which is known for its speed.

Advances in object detection using YOLO have an immense influence on ALPR system. Frequently, many authors adopted YOLO inspired models to improve performance [13,14]. A real-time object detector YOLOV2 model is employed in [15], and it is distinct for its speed and accuracy. A fast YOLO model [11] was employed in a cascaded manner and achieved a low recall rate. Using modified YOLO models, an LP detection was performed [16] and deployed to predict rotation angles of a LP [7]. A conclusive YOLO version is performed in [17], but shows a lack of accuracy on larger-sized objects. ALPR systems have been described in some reviews [18,19,20,21].

Based on the above, methods that gain a high FPS are due to modifications that are not in an overall ALPR system, such as skipping stages or only considering one vehicle per image. This might work well for specific application domains, but it is not appropriate for a general ALPR system.

## 3. Proposed Method

In this paper, we present a fully automated ALPR pipeline that does not use any pre-defined rules, together with the experimental findings associated with the YOLO detector. Each feature extraction model of our proposed system is expounded on in the following sub-sections and also explains the datasets used in detail.

This work is an improvement of the ALPR system explained in [12], even so, taking it towards a more generic automated ALPR system and utilize a better version of YOLO that makes bounding box coordinates and class probabilities directly from the image pixels [22]. This is because [12] have already achieved great results in five public datasets, demonstrated their results very clearly, and obtained a better recognition rate than most previous methods. They also leveraged post-processing rules to improve the recognition results. So here, we use the 5 datasets but with no post-processing or fixed rules based on country-specific layouts to ensure non-exclusive accuracy. Experiments were performed using the state-of-the-art YOLO v2 detector for the first stage and v4 detector for the remaining stages in the darknet framework [22] with a vehicle type classifier using ResNet50.

### 3.1. Datasets

Using more than one dataset is vital for a good overall ALPR system to obtain all kinds of different variations, such as lighting, backgrounds, vehicle sizes, and camera angles. Having an ALPR system that performs well in a wide range of datasets also means it will perform better in the real world. Most of the previous works focused on and showed their results based on a single dataset [8,10,11]. Using a few datasets makes the LPs biased towards those countries, and all the processing steps will be biased or will not provide enough substantial variations in the LP itself. Refs. [6,7,9] use a few datasets, but again, not quite enough, and most cases cover only one country. Ref. [12] uses 8 publicly available datasets, which contain significant variations between them, but Ref. [12] focuses on separating the ALPR system based on the country detected and has fixed rules at inference, which is not ideal for a fully automated ALPR as there are too many countries to cover. To ensure reproducibility, it is essential to keep the datasets publicly available, so that other researchers can make comparisons without any ambiguity about the parameters used. There are eight publicly available datasets that are common in the literature. In this paper, we use five publicly available datasets. The datasets used were Caltech Cars [23], English LP [24], OpenALPR EU [25], AOLP [26], and UFPR ALPR [27]. Table 1 shows key details of the datasets.

### 3.2. Proposed Framework

**Figure 1:**
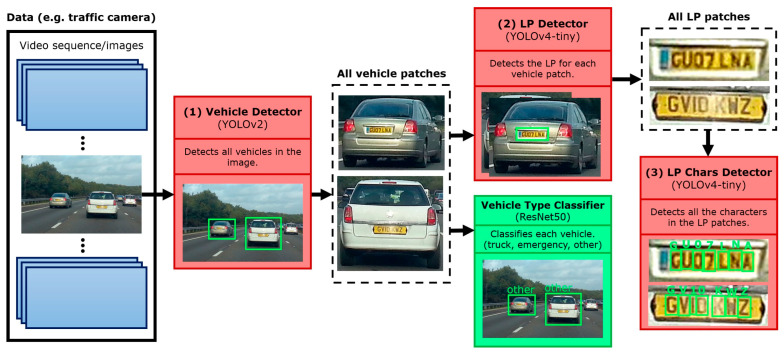
Illustration of the proposed framework.

All stages of the pipeline are made up of a YOLO detector [28], more specifically a YOLOv4 tiny detector [29] and a YOLO v2 for the first stage. YOLO was chosen because it is currently the state-of-the-art detector when it comes to speed without sacrificing too much accuracy. It is also used by many proposed methods in this domain because of its speed and desire to obtain real-time performance, such as [7,9,11,12]. However, all previous methods use old versions of YOLO, as YOLOv4 was recently published in 2020. In Figure 1, the full pipeline of our ALPR is illustrated.

The process starts with the full image, for example, from a surveillance camera which first goes through the vehicle detector stage (1), where all vehicles are detected. All the vehicle patches of the detected vehicles get cropped, and each of those patches then goes through two models. First, it goes through the vehicle classifier to determine what type of vehicle it is, for example, an emergency vehicle or a truck. Secondly, each vehicle patch also continues through to the LP detector (2), where the LP is detected. So now we would have an LP patch for each of the vehicles detected. All those LP patches then go through the final stage, the LP character detector (3), which is a detector that detects all characters in the LP patch. From there, the full LP characters are constructed. So, at the end, for every vehicle in the image, we will end up with the type of the vehicle (which can be extended to multiple classes, even to the vehicle make, model, year, etc. if needed) and the LP of the vehicle. An algorithmic representation of the proposed method can be seen in Algorithm 1.
**Algorithm 1:** ALP pipeline
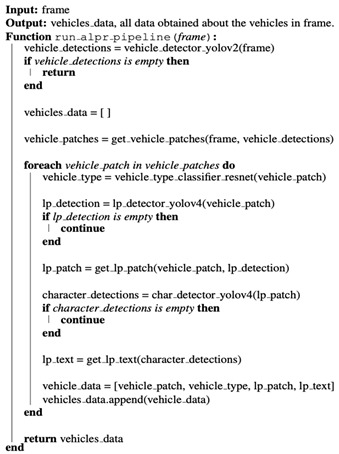


### 3.3. Vehicle Detection (VD)

For the vehicle detection, the same exact method as [12] was used. As a very high accuracy with excellent FPS was already achieved, there was not much improvement to be made in 99.92% recall when an intersection over union (IoU) of 0.25 was used. In addition, this method has a very high accuracy of 117 FPS. However, an addition was made to ignore all vehicle patches that were less than 40 pixels, either in width or height, as they would be too small for even a human to read the LP when enlarged. YOLOv2 is used for this stage; the model architecture is shown in Table 2.

### 3.4. Vehicle Type Classification (VTC)

For the vehicle type classification, as a start, a classifier was made for trucks and emergency vehicles, which could easily be expanded to other classes. There is also a third class, “other”, which refers to all other types of vehicles, for example, cars and motorcycles. Images were gathered from random places on the internet, including images from Open Images V6 [30]; 449 samples of emergency vehicles, 374 samples of trucks, and 831 samples of “other” were used. The classifier used is a ResNet50 [31] with data augmentations of rotation, width and height shifts, brightness, shear, zoom, and horizontal flip. Transfer learning was used where the weights used are from the ResNet50 model trained on the COCO dataset [32]. All the ResNet50 layers are frozen, and average pooling was added to the end, followed by a fully connected layer of 32, followed by the SoftMax output layer, which led to 65,667 trainable parameters with 23,587,712 frozen weights that were trained on the COCO dataset.

### 3.5. License Plate Detection (LPD)

The LPD stage is a YOLOv4-tiny model. The training images are the cropped vehicle patches from the full images. To increase the dataset and improve accuracy, the dataset was doubled using the negative images of all samples; this doubling of the dataset increased the accuracy significantly. The detection is constrained to detections with an IoU of greater than 0.65, and if multiple were detected, the highest confidence score was chosen, as a vehicle can only have one LP. The full model architecture can be seen in Table 3. By using the latest YOLOv4 detector, we will see in Section 4 that this detector outperforms previous methods in the same dataset.

### 3.6. License Plate Recognition (LPR)

The first two stages are much easier; they do not bring any major challenges, and with enough data, it is fairly easy to get high accuracy. The real challenge is the final stage, where you must obtain each character of the LP to ultimately identify the vehicle. 

In this stage, again, the YOLOv4 tiny detector was used. Table 4 shows the full LPR YOLO network architecture. There were no major modifications done to the architecture apart from: (1) changing the network input to 352 × 128, which was chosen because the average aspect ratio (w/h) of all LP patches across all datasets is 2.86; (2) the number of filters in each convolutional layer before each YOLO layer was filters = ((classes + 5) × anchors), where the number of classes is 36 (0–9 + A–Z) and the number of anchors is 3, resulting in 123 filters; (3) disabled the flip augmentation, as this will result in flipped characters, which will not be useful for the model to learn. Only LP patches that are larger than 20 and 10 pixels in width and height, respectively, were considered to be LPs.

It is important to note that all digits and characters were considered their own class, so the classes were 0–9, and A–Z, making a total of 36 classes. Unlike other methods such as [6,11,12], where, for example, the digit “0” was assumed to be the same class as the letter “O”. Then, using this coupled with the fixed rules of the LP character sequence, they would choose whether it is a zero or “O” after the predictions have happened based on how the LP characters are sequenced for that country in the dataset. For example, if the first three characters of an LP in a certain country are said to always be letters, then any digit zero predicted for the first three characters will be classified as the letter “O”. However, as discussed earlier, having such fixed rules is not ideal and will breakdown in certain cases, such as foreign or custom LPs; it is not generalized. So here, all digits and letters are considered in the alphabet as having their own specific class. This allowed for no fixed post-processing at all during inference and allowed for a general ALPR system.

From the early baseline experiments carried out, it was found out that there were only a few characters that were performing poorly, specifically, characters with an average precision (AP) below 0.95. The low-performing characters (LPC) were all letters, and they were “G”, “K”, “M”, “O”, “Q”, and “S”. To increase the AP of the LPC, the number of samples that include any LPC was increased by using data permutations and data generation.

#### 3.6.1. Data Permutations

**Figure 2:**
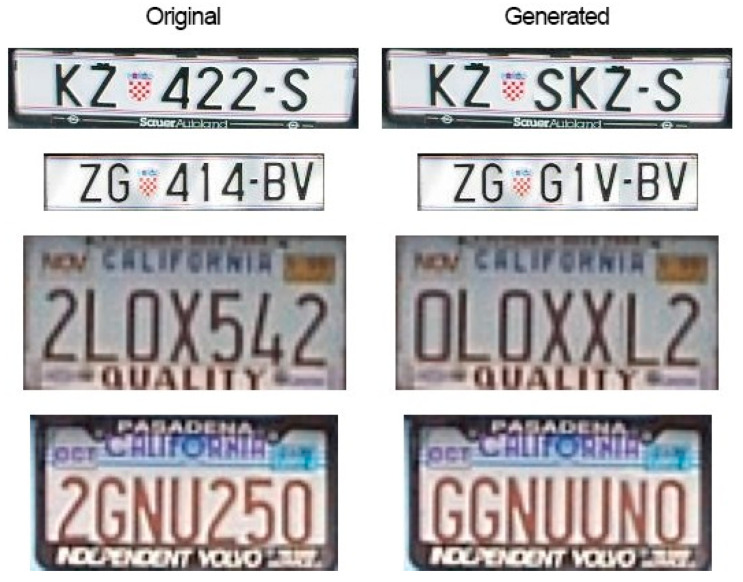
Data generation using permutations.

In this method, every LP that contained any of the LPC was duplicated by replacing other numbers or other letters that were not the LPC with LPC characters. This is illustrated in Figure 2. 

This is done by using the annotated BB of each character and replacing it with the corresponding LPC patch. The digit one was not replaced as it made the patches of the other letters resize into a narrow vertical patch that would distort the character, which caused the model to get confused and perform poorly.

#### 3.6.2. Data Generation

**Figure 3:**
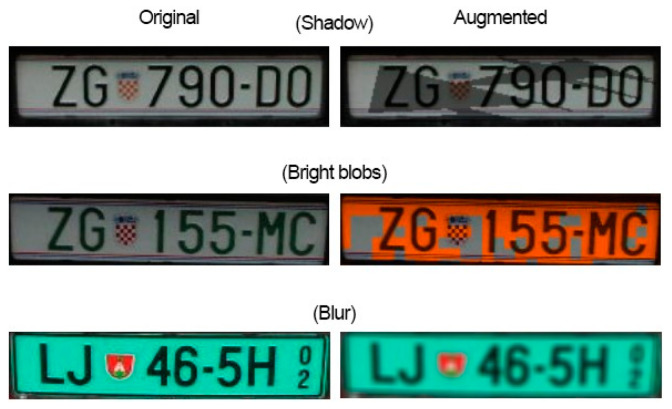
Data generation techniques on different license plates.

With data generation, all LP samples that contained the LPC were doubled by using three different augmentation techniques to imitate certain natural changes that might happen to the LP under certain circumstances. This is illustrated in Figure 3. 

The first is generating an artificial shadow and placing it randomly over the LP patch. This will change the overall look of some of the characters, and this situation can also be met in practice. The second is adding a color that is similar to the sun, but more importantly, adding a variation to the LP that will force the model to learn to ignore it making it more generalizable for real-world scenarios. The third method is adding random blur, which is to replicate bad camera angles, speeding vehicles, etc. So, for each LP patch sample that included the LPC, each of these three techniques had an equal chance of being applied. Using these two methods increased the number of samples by 2381, and all those samples included the LPC, which were in addition to the doubling of samples from using the negative image of each sample, making a total of 16,961 samples.

**Figure 4:**
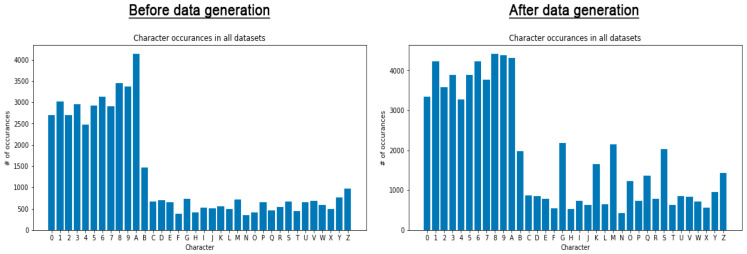
The total number of character occurrences for all datasets before and after data generation.

Figure 4 shows two histograms of all the character counts before and after the data generation. We can clearly see that each LPC has a significant increase in the number of total characters, giving the model more samples to train on and hence improving the accuracy for those characters. This turned out to be very useful, as we will see in Section 4.

## 4. Results

Since some papers only partially considered the ALPR pipeline, each stage will be evaluated and compared separately in this section. Only the UFPR ALPR dataset had fixed training, validation, and testing sets. For the rest of the datasets, they were split using 0.7, 0.2, and 0.1 ratios for training, validation, and testing sets, respectively. To ensure there was no bias in selecting the sets, each experiment was carried out using five different random splits. All results are obtained using the test sets average results across the five splits. The evaluation metrics commonly used in object detection are precision [7,8] and recall, and they can be written as:

$$\mathrm{Precision}\;=\frac{\mathrm{TP}\;}{\mathrm{TP}}+\mathrm{FP}$$

$$\mathrm{Recall}=\frac{\mathrm{TP}}{\mathrm{TP}}+\mathrm{FN}$$

where TP is the metric defined based on ground truth correctly labeled as positives, FP is negative examples incorrectly labelled as positives, FN is a false negative, and TN refers to the examples correctly labelled as negatives.

To predict the quality of object detection, we use the Jaccard index called Intersection over Union (IoU), which can be expressed as

$$\mathrm{IoU}=\frac{\mathrm{area}\left(B_{p}\cap B_{\mathrm{gt}}\right)}{\mathrm{area}\left(B_{p}\cup B_{\mathrm{gt}}\right)}$$

where B_P_ is the predicted bounding box and B_gt_ is ground truth bounding box.

### 4.1. Vehicle Detection Results

As we can see from Table 5, the VD stage is not an issue; a very impressive accuracy can be achieved across all datasets and is not open to much improvement. What should be noted, is that the slight precision loss is only due to false positive vehicles detected that did not have an LP visible (apart from AOLP). So, all vehicles that should have been detected were detected, just with some extra vehicles detected in the background, which did not have an LP visible. A confidence and IoU threshold of 0.5 and 0.25 were used, respectively.

### 4.2. Vehicle Type Classification Results

**Figure 5:**
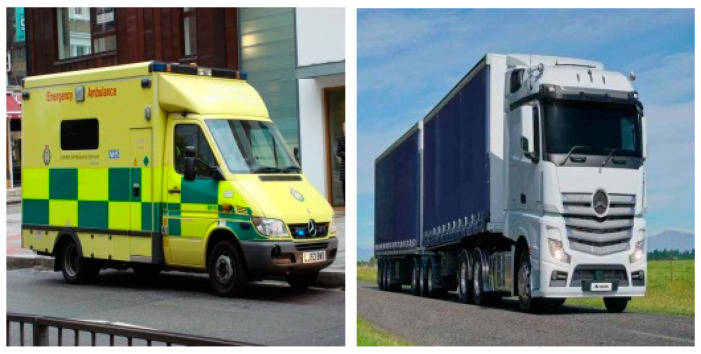
Sample images of an emergency vehicle and a truck.

Trucks and emergency vehicles are just used as example classes; this stage can be expanded to many more classes and data, even identifying, for example, vehicle make, model, year, color, etc. In Figure 5, we can see some samples of the two main classes.

In Table 6, the results are shown. All samples used are made public and can be accessed through the repository. The results are all from the average test sets across the five different splits. As we can see, a very high accuracy of 98.22% is achieved. This stage can easily be expanded by just adding more annotations to the samples.

### 4.3. LP Detection Results

In Table 7, we can see that the average recall for the LP detection stage across all datasets is above 99%, which is certainly an acceptable performance. A confidence threshold of 0.75 and an IoU threshold of 0.5 were used.

As we can see from the results, the first two stages of the ALPR are not a problem, and very high accuracy can be consistently achieved throughout multiple datasets. However, there could be improvements to the average IoU.

### 4.4. LP Recognition Results

In this section, we will focus specifically on the results of the last stage, the LPR stage, skipping the first two stages to isolate this stage. In the next section, we will see the results of the full ALPR pipeline. So here, all LP patches from all datasets are considered; this is to evaluate only the LP recognition stage.

Table 8 shows the results; a confidence threshold of 0.75 and IoU threshold of 0.5 were used. We can see that we get pretty impressive results across all datasets, apart from the UFPR ALPR dataset; the reason why this is the case will be discussed in the next section.

### 4.5. Full ALPR Pipeline Results

Here we see the results of the full ALPR pipeline. A correct LP recognition is only considered if all stages in the pipeline were successful and all LP characters are correctly detected. So, if, for example, a vehicle or an LP is not detected and does not go to the next stage(s), it is considered a wrong sample.

As we can see from Table 9, without any post-processing to the LP patches or any fixed rules based on prior knowledge of the LPs after predictions, highly accurate results are achieved across all datasets. Each crop at each stage is directly fed into the next stage the same way it was detected. Note that for the Caltech cars, English LP, AOLP, and Open ALPR EU, we have a relatively small number of FN, and in some cases where the test set is so small, this makes a huge impact on the final recall when it really is just one vehicle or LP that is not detected correctly.

The UFPR ALPR is clearly the more challenging dataset; however, it has to be noted that the UFPR ALPR dataset is made up of images from a video. For example, you would have 30 images from the same video with slight differences as the vehicle is moving. The UFPR ALPR test set is made up of 60 videos, with each having a total of 30 frames. In practice, when you have a video stream like that, you will have many different opportunities (frames) to correctly detect the full LP as the car moves across the frame, and you can, for example, consider a recognition with 100% confidence if the same LP for the same vehicle has been detected three times in a row (3 frames in a row). That is, the rest of the frames in which the vehicle is present is not significant as you only need as little as three consecutive frames for the LP to be successfully recognized. If we treat the UFPR ALPR dataset in this way, as in practical settings, from the bottom of Table 9 (UFPR ALPR as vid), we can see the recognition result is significantly higher as we do not need to detect the LP of the vehicle on every frame if we have already detected it correctly 3 times in 3 consecutive frames.

We can see that the full ALPR pipeline, all stages, are performing very well, and while the final stage is clearly the least performing, it is still producing very good results without assuming any prior knowledge on the LP across all datasets and considering all characters of the alphabet as their own class.

### 4.6. Comparison

In this section, we compare the results of proposed systems with the results of this work across five datasets. Table 10 summarizes all the results.

As we can see, without any processing at inference or relying on any fixed rules in a streamlined pipeline, similar and comparable results are achieved while still considering all characters as their own class, 0–9 and A–Z. Table 11 shows the results for each character.

We can clearly see that there are only a few characters that are performing poorly (AP less than 95), which are the “K”, “M”, “O”, and “Q” characters, and for those letters, it is very understandable why they would be very hard and challenging for a model to distinguish between. However, with more data and more data generation techniques, the accuracy of these characters can be increased, and a full generic ALPR system can be achieved.

**Figure 6:**
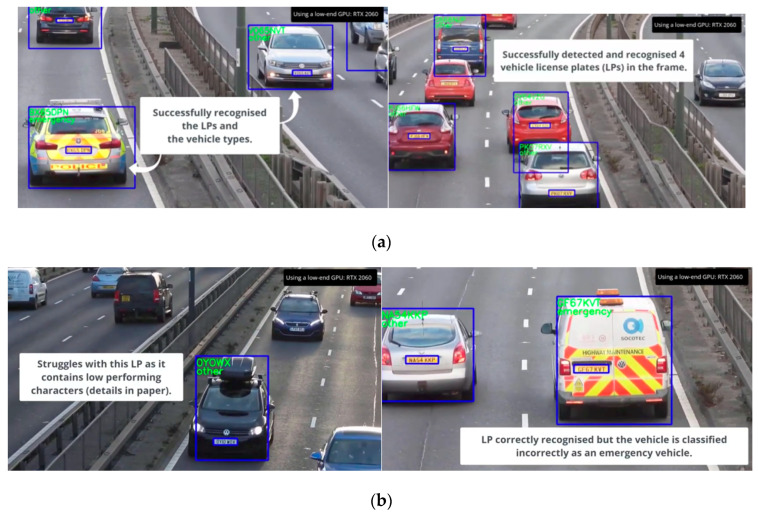
(**a**) Examples of successful detection of LPs and vehicle types; (**b**) Examples of images in which LP detection or classification failed.

A video demonstration of our method using unseen data is available at https://youtu.be/aZFHGMyllAI (accessed on 27 October 2022) for better understanding. Figure 6 shows a few example images when processing it in real time.

### 4.7. Performance Evaluation

Table 12 shows the time it takes to perform each stage in seconds when there are 1, 2, and 3 vehicles in the frame where the LP is visible. It also shows the total FPS it takes to process the whole frame for all stages. It has to be noted that these performance results are not a fair comparison to other methods because a very low-end GPU (NVIDIA GTX 1060) was used in these experiments, whereas most other methods used a very high-end, expensive GPU. However, the fact that we achieve this FPS even when using a low-end GPU shows promising results in terms of performance. We believe that if the same high-end GPUs were used, this ALPR pipeline could easily achieve real-time performance.

## 5. Conclusions

We have presented a method to perform the ALPR task in a fully automated and stream-lined pipeline that includes all three stages, including a vehicle classifier, without exploiting any prior knowledge of the LP, or utilizing fixed pre/post processing rules. This establishes the generalizability and suitability of the proposed method in a real-time context. We performed our experiments in a darknet framework using the YOLOv4 detector. Despite the positions, YOLO has remarkable versatility in learning features, and we tune parameters separately at each stage to improve the performance.

Data augmentation techniques were performed to deal with illumination and artifacts, and multiple data generation techniques were developed to increase data samples. Competitive results were achieved when compared to previous methods tackling the full ALPR problem. Our method shows promising results in terms of accuracy and performance on five different publicly available datasets. Only a few characters are left that are poorly detected; with slightly more data, a very robust and general ALPR system is possible. This method is a step towards a complete and fully automated ALPR system that works with any type of LP regardless of where it is from or its layout, which will be essential in a complete future ITS system. We have also made this methodology open source to use and/or contribute to. The full implementation of the ALPR pipeline is openly available at: https://github.com/RedaAlb/alpr-pipeline (accessed on 27 October 2022) and it allows researchers to compare novel approaches. In our future work, we intend to investigate the possibilities of advancements in the ALPR system using latest versions of YOLO. We intend to explore the possibilities of a more robust model in real-world applications and investigate the challenges of contemporaneous events.

## Figures and Tables

**Table 1:** An overview of the datasets used in our experiments and its specifications.

Dataset	Resolution	Country	Year	# Samples
Caltech Cars	896 × 592	America	1999	124
English LP	640 × 480	EU	2003	509
OpenALPR EU	diverse	EU	2016	108
AOLP	diverse	Taiwan	2013	2049
UFPR ALPR	1920 × 1080	Brazil	2018	4500
Total Samples				7290

**Table 2:** Vehicle detection model architecture.

Layer	Filters	Size/Strd	Input	Output
0 conv	32	3 × 3/1	608 × 416 × 3	608 × 416 × 32
1 max		2 × 2/2	608 × 416 × 32	304 × 208 × 32
2 conv	64	3 × 3/1	304 × 208 × 32	304 × 208 × 64
3 max		2 × 2/2	304 × 208 × 64	152 × 104 × 64
4 conv	128	3 × 3/1	152 × 104 × 64	152 × 104 × 128
5 conv	64	1 × 1/1	152 × 104 × 128	152 × 104 × 64
6 conv	128	3 × 3/1	152 × 104 × 64	152 × 104 × 128
7 max		2 × 2/2	152 × 104 × 128	76 × 52 × 128
8 conv	256	3 × 3/1	76 × 52 × 128	76 × 52 × 256
9 conv	128	1 × 1/1	76 × 52 × 256	76 × 52 × 128
10 conv	256	3 × 3/1	76 × 52 × 128	76 × 52 × 256
11 max		2 × 2/2	76 × 52 × 256	38 × 26 × 256
12 conv	512	3 × 3/1	38 × 26 × 256	38 × 26 × 512
13 conv	256	1 × 1/1	38 × 26 × 512	38 × 26 × 256
14 conv	512	3 × 3/1	38 × 26 × 256	38 × 26 × 512
15 conv	256	1 × 1/1	38 × 26 × 512	38 × 26 × 256
16 conv	512	3 × 3/1	38 × 26 × 256	38 × 26 × 512
17 max		2 × 2/2	38 × 26 × 512	19 × 13 × 512
18 conv	1024	3 × 3/1	19 × 13 × 512	19 × 13 × 1024
19 conv	512	1 × 1/1	19 × 13 × 1024	19 × 13 × 512
20 conv	1024	3 × 3/1	19 × 13 × 512	19 × 13 × 1024
21 conv	512	1 × 1/1	19 × 13 × 1024	19 × 13 × 512
22 conv	1024	3 × 3/1	19 × 13 × 512	19 × 13 × 1024
23 conv	1024	3 × 3/1	19 × 13 × 1024	19 × 13 × 1024
24 conv	1024	3 × 3/1	19 × 13 × 1024	19 × 13 × 1024
25 route	16			38 × 26 × 512
26 reorg		/2	38 × 26 × 512	19 × 13 × 2048
27 route	26–24			19 × 13 × 3072
28 conv	1024	3 × 3/1	19 × 13 × 3072	19 × 13 × 1024
29 conv	35	1 × 1/1	19 × 13 × 1024	19 × 13 × 35

**Table 3:** LP detection model architecture.

Layer	Filters	Size/Strd	Input	Output
0 conv	32	3 × 3/2	416 × 416 × 3	208 × 208 × 32
1 conv	64	3 × 3/2	208 × 208 × 32	104 × 104 × 64
2 conv	64	3 × 3/1	104 × 104 × 64	104 × 104 × 64
3 route	2		1/2	104 × 104 × 32
4 conv	32	3 × 3/1	104 × 104 × 32	104 × 104 × 32
5 conv	32	3 × 3/1	104 × 104 × 32	104 × 104 × 32
6 route	54			104 × 104 × 64
7 conv	64	1 × 1/1	104 × 104 × 64	104 × 104 × 64
8 route	27			104 × 104 × 128
9 max		2 × 2/2	104 × 104 × 128	52 × 52 × 128
10 conv	128	3 × 3/1	52 × 52 × 128	52 × 52 × 128
11 route	10		1/2	52 × 52 × 64
12 conv	64	3 × 3/1	52 × 52 × 64	52 × 52 × 64
13 conv	64	3 × 3/1	52 × 52 × 64	52 × 52 × 64
14 route	13–12			52 × 52 × 128
15 conv	128	1 × 1/1	52 × 52 × 128	52 × 52 × 128
16 route	10–15			52 × 52 × 256
17 max		2 × 2/2	52 × 52 × 256	26 × 26 × 256
18 conv	256	3 × 3/1	26 × 26 × 256	26 × 26 × 256
19 route	18		1/2	26 × 26 × 128
20 conv	128	3 × 3/1	26 × 26 × 128	26 × 26 × 128
21 conv	128	3 × 3/1	26 × 26 × 128	26 × 26 × 128
22 route	21–20			26 × 26 × 256
23 conv	256	1 × 1/1	26 × 26 × 256	26 × 26 × 256
24 route	18–23			26 × 26 × 512
25 max		2 × 2/2	26 × 26 × 512	13 × 13 × 512
26 conv	512	3 × 3/1	13 × 13 × 512	13 × 13 × 512
27 conv	256	1 × 1/1	13 × 13 × 512	13 × 13 × 256
28 conv	512	3 × 3/1	13 × 13 × 256	13 × 13 × 512
29 conv	18	1 × 1/1	13 × 13 × 512	13 × 13 × 18
30 yolo				
31 route	27			13 × 13 × 256
32 conv	128	1 × 1/1	13 × 13 × 256	13 × 13 × 128
33 up	2x		13 × 13 × 128	26 × 26 × 128
34 route	33–23			26 × 26 × 384
35 conv	256	3 × 3/1	26 × 26 × 384	26 × 26 × 256
36 conv	18	1 × 1/1	26 × 26 × 256	26 × 26 × 18
37 yolo				

**Table 4:** LP recognition model architecture.

Layer	Filters	Size/Strd	Input	Output
0 conv	32	3 × 3/2	352 × 128 × 3	176 × 64 × 32
1 conv	64	3 × 3/2	176 × 64 × 32	88 × 32 × 64
2 conv	64	3 × 3/1	88 × 32 × 64	88 × 32 × 64
3 route	2		1/2	88 × 32 × 32
4 conv	32	3 × 3/1	88 × 32 × 32	88 × 32 × 32
5 conv	32	3 × 3/1	88 × 32 × 32	88 × 32 × 32
6 route	54			88 × 32 × 64
7 conv	64	1 × 1/1	88 × 32 × 64	88 × 32 × 64
8 route	27			88 × 32 × 128
9 max	0	2 × 2/2	88 × 32 × 128	44 × 16 × 128
10 conv	128	3 × 3/1	44 × 16 × 128	44 × 16 × 128
11 route	10		1/2	44 × 16 × 64
12 conv	64	3 × 3/1	44 × 16 × 64	44 × 16 × 64
13 conv	64	3 × 3/1	44 × 16 × 64	44 × 16 × 64
14 route	13–12			44 × 16 × 128
15 conv	128	1 × 1/1	44 × 16 × 128	44 × 16 × 128
16 route	10–15			44 × 16 × 256
17 max		2 × 2/2	44 × 16 × 256	22 × 8 × 256
18 conv	256	3 × 3/1	22 × 8 × 256	22 × 8 × 256
19 route	18		1/2	22 × 8 × 128
20 conv	128	3 × 3/1	22 × 8 × 128	22 × 8 × 128
21 conv	128	3 × 3/1	22 × 8 × 128	22 × 8 × 128
22 route	21–20			22 × 8 × 256
23 conv	256	1 × 1/1	22 × 8 × 256	22 × 8 × 256
24 route	18–23			22 × 8 × 512
25 max		2 × 2/2	22 × 8 × 512	11 × 4 × 512
26 conv	512	3 × 3/1	11 × 4 × 512	11 × 4 × 512
27 conv	256	1 × 1/1	11 × 4 × 512	11 × 4 × 256
28 conv	512	3 × 3/1	11 × 4 × 256	11 × 4 × 512
29 conv	123	1 × 1/1	11 × 4 × 512	11 × 4 × 123
30 yolo				
31 route	27			11 × 4 × 256
32 conv	128	1 × 1/1	11 × 4 × 256	11 × 4 × 128
33 up		2×	11 × 4 × 128	22 × 8 × 128
34 route	33–23			22 × 8 × 384
35 conv	256	3 × 3/1	22 × 8 × 384	22 × 8 × 256
36 conv	123	1 × 1/1	22 × 8 × 256	22 × 8 × 123
37 yolo				

**Table 5:** Results attained in vehicle detection (%) and the recall rates achieved in all datasets.

Dataset	Precision	Recall	Avg IoU
Caltech cars	100.00	100.00	96.84
English LP	99.88	100.00	94.98
AOLP	98.96	99.52	94.27
Open ALPR EU	100.00	100.00	95.30
UFPR ALPR	99.50	100.00	90.35
Average	99.71	99.90	94.35

**Table 6:** Vehicle type classification test set results.

Accuracy (%)	Loss
98.22	0.1130

**Table 7:** LP detection results (%) in all five datasets.

Dataset	Precision	Recall	Avg IoU
Caltech cars	100.00	99.19	86.72
English LP	99.61	99.21	83.70
AOLP	99.43	99.67	86.26
Open ALPR EU	100.00	99.07	85.54
UFPR ALPR	96.78	98.67	83.52
Average	99.16	99.36	85.15

**Table 8:** LP recognition results without the first two stages (%).

Dataset	Precision	Recall	Avg IoU
Caltech cars	100.00	98.98	90.42
English LP	99.91	99.87	93.16
AOLP	99.94	99.87	89.38
Open ALPR EU	100.00	98.66	91.30
UFPR ALPR	98.57	91.08	85.57
Average	99.68	97.69	89.97

**Table 9:** Full ALPR pipeline results. Please note these results are chosen from one of the five test sets just to include the exact TP and FN to demonstrate how significant just one incorrect sample in the relatively small test sets can be in some datasets. All the other test sets had very similar results.

Dataset	Stage	TP	FN	Recall
	VD	14	0	100
Caltech cars	LPD LPR	13 13	1 1	92.86 92.86
	VD	52	0	100
English LP	LPD LPR	50 50	2 2	96.15 96.15
	VD	218	1	99.54
AOLP	LPD LPR	216 214	3 5	98.63 97.72
	VD	12	0	100
Open ALPR	LPD LPR	12 12	0 0	100 100
	VD	1800	0	100
UFPR ALPR	LPD LPR	1769 1117	31 683	98.28 62.06
UFPR ALPR as vid	LPR	44	16	73.33
Average LPR				89.56

**Table 10:** ALPR comparison to other methods across all datasets used. * The first two stages of the pipeline were skipped. ** When considering the UFPR ALPR dataset as a video.

Method	[33]	[5]	[34]	[35]	[36]	OpenALPR	[12]	Proposed
Dataset								
Caltech cars	-	-	-	-	95.7 ± 2.7	99.1 *±* 1.2	98.7 *±* 1.2	97.1
English LP	97.0	-	-	-	92.5 ± 3.7	78.6 ± 3.6	95.7 ± 2.3	95.5
AOLP	-	99.8 *	-	-	87.1 ± 0.8	-	99.2 ± 0.4	98.0
Open ALPR EU	-	-	93.5	85.2	93.5	91.7	97.8 ± 0.5	98.7
UFPR ALPR	-	-	-	-	62.3	82.2	90.0 ± 0.7	62.1 (73.3 **)
Average	-	-	-	-	87.8 ± 2.4	90.7 ± 2.3	96.9 ± 1.0	90.3

**Table 11:** All characters’ average precision (AP) across all datasets in the test sets combined.

C	AP (%) TP	FP	C	AP (%) TP	FP
**0**	98.26 724	22	**I**	98.90 94	0
**1**	99.65 844	4	**J**	100.00 145	0
**2**	100.00 497	0	**K**	83.86 115	0
**3**	99.51 645	0	**L**	100.00 146	0
**4**	99.93 811	16	**M**	86.50 165	23
**5**	100.00 758	0	**N**	100.00 48	0
**6**	99.94 922	6	**O**	38.96 35	28
**7**	99.17 669	15	**P**	99.99 282	0
**8**	98.72 1037	15	**Q**	70.84 43	1
**9**	99.66 1103	8	**R**	99.98 148	2
**A**	98.82 1494	2	**S**	96.47 228	0
**B**	97.98 363	0	**T**	100.00 112	0
**C**	97.64 133	3	**U**	100.00 128	0
**D**	99.53 99	13	**V**	100.00 171	0
**E**	97.59 136	6	**W**	95.70 229	1
**F**	99.06 40	1	**X**	98.69 107	0
**G**	95.68 147	0	**Y**	99.95 274	0
**H**	99.96 109	0	**Z**	100.00 280	0

**Table 12:** The FPS for the full ALPR pipeline, as well as the processing time in seconds for each stage of the pipeline when there are 1, 2, and 3 vehicles in the frame.

Vehicles Stage	1	2	3
Vehicle detection	0.0349	0.0389	0.0449
LP detection	0.0080	0.0150	0.0239
LP recognition	0.0120	0.0239	0.0239
Total FPS	18	13	11

## Data Availability

All data generated or appeared in this study are available upon requested by contact with the corresponding author. Moreover, the models and code used during the study can be found on github. https://github.com/RedaAlb/alpr-pipeline (accessed on 27 October 2022).
